# Structure-guided drug repurposing identifies aristospan as a potential inhibitor of β-lactamase: insights from virtual screening and molecular dynamics simulations

**DOI:** 10.3389/fphar.2024.1459822

**Published:** 2024-11-06

**Authors:** Moyad Shahwan, Mohd Shahnawaz Khan, Azna Zuberi, Nojood Altwaijry, Anas Shamsi

**Affiliations:** ^1^ Center for Medical and Bio-Allied Health Sciences Research, Ajman University, Ajman, United Arab Emirates; ^2^ College of Pharmacy and Health Sciences, Ajman University, Ajman, United Arab Emirates; ^3^ Department of Biochemistry, College of Science, King Saud University, Riyadh, Saudi Arabia; ^4^ Division of Reproductive Science in Medicine, Department of Obstetrics and Gynecology, Feinberg School of Medicine, Northwestern University, Chicago, IL, United States

**Keywords:** β-lactamase, antibiotic resistance, drug repurposing, virtual screening, molecular dynamics simulation, SM23, Aristospan

## Abstract

The rise of β-Lactamase mediated antibiotic resistance is a major concern for public health; hence, there is an urgent need to find new treatment approaches. Structure-guided drug repurposing offers a promising approach to swiftly deliver essential therapeutics in the fight against escalating antibiotic resistance. Here, a structure-guided virtual screening approach was used involving drug profiling, molecular docking, and molecular dynamics (MD) simulation to identify existing drugs against β-Lactamase-associated drug resistance. We exploited a large panel of FDA-approved drugs to an extensive *in silico* analysis to ascertain their ability to inhibit β-Lactamase. First, molecular docking investigations were performed to assess the binding affinities and interactions of screened molecules with the active site of β-Lactamase enzymes. Out of all the screened candidates, Aristospan was identified to possess promising characteristics, which include appropriate drug profiles, high binding specificity, and efficiency towards the binding pocket of β-Lactamase. Further analysis showed that Aristospan possesses several desirable biological characteristics and tends to bind to the β-Lactamase binding site. To explore the interactions further, the best docking pose of Aristospan was selected for MD simulations to assess the thermodynamic stability of the drug-enzyme complex and its conformational changes over 500 ns. The MD simulations in independent replica runs demonstrated that the β-Lactamase-Aristospan complex was stable in the 500 ns trajectory. These enlightening results suggest that Aristospan may harbor the potential for further evolution into a possible β-Lactamase inhibitor, with potential applications in overcoming antibiotic resistance in both Gram-positive and Gram-negative bacteria.

## 1 Introduction

Antibiotic resistance has appeared as one of the most significant problems in the modern world and the healthcare system (Laxminarayan et al., 2013). The development and dissemination of β-lactamase-mediated resistance occur at an alarming rate, thus affecting the effectiveness of the beta-lactam antibiotics, which are highly utilized in antibacterial therapy (Bush and Bradford, 2020). β-lactamases are enzymes produced by a variety of bacterial pathogens, including staphylococci, and these enzymes cleave the β-lactam ring in the antibiotics, thus making them inactive (Alfei and Schito, 2022). This progressive increase in resistance calls for novel approaches to inhibit these enzymes and effectively enhance the efficacy of β-lactam antibiotics (Lima et al., 2020).

Classically, drug discovery has been based on the experimental identification of molecules with new chemical formulations and modes of action (Swinney and Anthony, 2011). However, this approach is challenging due to high failures, a long time to develop, and increasing costs (Schenone et al., 2013). In addressing these challenges, repurposing existing drugs has proved to be a viable solution (Park, 2019). Repurposing builds on the known information about the safety, pharmacokinetics, and pharmacodynamics of already approved drugs to utilize the same drugs for other diseases, thereby shortening the time taken to get new chemical entities to the clinic (Parvathaneni et al., 2019). By employing this approach, it becomes possible to make the best out of the existing chemical data to meet the unmet health requirements more effectively.

Bioinformatics approaches have impacted the drug discovery process by altering how the process is approached (Rastogi et al., 2022). The introduction of molecular docking and molecular dynamics (MD) simulation in the drug discovery process has led to better drug design strategies (Mohammad et al., 2020a). Molecular docking is useful in determining the possible binding sites and the corresponding binding energies of small molecule ligands to the protein target, which is useful in selecting good drug candidates (Basu et al., 2020). After that, the MD simulations enable the detailed examination of the dynamic properties of the protein-ligand complex. When combined, molecular docking and MD simulations have demonstrated their effectiveness as powerful tools that significantly reduce the time required for drug discovery (Naqvi et al., 2018).

Concerning β-lactamase targeting, this study aims to exploit advanced *in silico* applications for therapeutic advancements. Our goal is to repurpose potential FDA-approved drugs for β-lactamase inhibition based on a rigorous bioinformatics analysis. The advantage of using repurposed drugs as potential inhibitors is that they have already been through the safety evaluation process, which means that they have been approved for use in other capacities; thus the process of getting them to the clinic should not be as rigid as it is for other new drugs. In this study, we combined molecular docking and MD simulation to estimate the binding potential, interaction modes, and dynamic profiles of various repurposed small molecules against β-lactamase. This methodology seeks to identify lead-like compounds capable of interacting with the binding site of β-lactamase and thereby inhibiting its function.

Our study commenced with a broad-spectrum *in silico* screening with molecular docking to evaluate the binding affinities and interactions of repurposed molecules with the active site of β-lactamase. The best hits from the docking analysis were subsequently selected for MD simulation to explore the stability and dynamic properties of the complexes formed between the selected drug molecules and β-lactamase across the time scale. Out of the screened candidates, Aristospan showed a quite appropriate drug profile, pharmacokinetic features, and binding potential. Aristospan also proved to have a high binding affinity, selectivity, and potency towards the β-lactamase binding site. To obtain detailed insights into the interactions of Aristospan-β-lactamase along with the reference inhibitor-protein complex and their conformational changes, we performed MD simulations for 500 nanoseconds (ns). These all-atom simulations offered detailed insights into the dynamics and interaction mechanism of the β-lactamase with the selected compounds.

## 2 Materials and methods

### 2.1 Data Preparation: β-Lactamase and FDA-Approved drugs

A well-established bioinformatics protocol for structure-guided discovery was followed on a DELL Z840 workstation. Various computational software such as AutoDock Tools (Huey et al., 2012), InstaDock v1.2 (Mohammad et al., 2021), PyMOL (DeLano, 2002), BIOVIA Discovery Studio Visualizer 4.5 (Visualizer, 2005), and GROMACS 2020 beta (Van Der Spoel et al., 2005) were used for performing virtual screening and all-atom MD simulations. The three-dimensional crystal structure of the *Escherichia coli* β-Lactamase protein was downloaded from the Protein Data Bank (PDB ID: 5CHJ). For docking, the initial protein structure was checked for any missing atoms and solvent molecules and was cleaned to remove any unnecessary ligands. The β-Lactamase structure was minimized using the energy minimization function in the Swiss-PDB Viewer tool (Kaplan and Littlejohn, 2001). A set of 3,648 FDA-approved drugs was acquired from DrugBank (Knox et al., 2024) and refined for docking simulations. Each compound’s 3D structure was analyzed and further processed through InstaDock and AutoDock tools, ensuring proper ionization state and tautomeric form.

### 2.2 Molecular docking screening

We conducted a molecular docking screening to identify molecules with high binding potential for β-Lactamase. The docking studies were carried out using InstaDock, an easy-to-use and widely recognized virtual screening software. A grid encompassing the entire β-Lactamase was generated using blind docking parameters, with dimensions: *X*-axis = 71 Å, *Y*-axis = 64 Å, and *Z*-axis = 68 Å. The grid was centered at X: 74.252 Å, Y: −10.248 Å, and Z: 18.469 Å with a spacing of 1 Å. All other docking parameters were kept at their default values. The standard virtual screening protocol of InstaDock was applied to initially screen the entire dataset of FDA-approved drugs.

### 2.3 Retrospective docking validation

The reliability of the docking procedure was verified through retrospective docking (Sousa et al., 2013). This evaluation included redocking a co-crystallized reference inhibitor, SM23, with β-Lactamase and comparing the resulting docked poses with the original co-crystallized poses. The validation results confirmed the successful and accurate execution of our docking procedure. Specifically, the docking method consistently predicted a binding pose for SM23 that quite matched the co-crystallized pose of SM23 on β-Lactamase (PDB ID: 5CHJ). These binding poses were illustrated in Supplementary Figure S1, where the docked SM23 and the crystallographically derived SM23 were compared. The strong alignment between the docked and co-crystallized SM23 orientations provides convincing confirmation for the robustness and efficiency of the docking procedure.

### 2.4 Drug profiling and PASS analysis

The biological activities of the screened molecules were explored in the available literature for drug profiling and analyzed using the online PASS program (Filimonov et al., 2014). This program allows for the examination of the potential biological activities of compounds on the basis of their chemical structures. It uses molecular fragment descriptors of the compounds, which suggest their biological properties based on spatial dispositions. The program offers a biological activity score, which is a probability ratio of ‘probability to be active (Pa)’ to ‘probability to be inactive (Pi)’. The higher the Pa value, the higher the chance that compounds will possess a particular biological property.

### 2.5 Scoring and interaction analysis

Following the drug profiling and PASS analysis, the selected compounds underwent interaction analysis. The top-ranked hits were visualized to evaluate their binding prototype with β-Lactamase using PyMOL and Discovery Studio Visualizer tools. This analysis of molecular docking and interactions led to the identification of repurposed molecules that exhibited strong binding potential and favorable interactions towards β-Lactamase.

### 2.6 MD simulations

Molecular dynamics (MD) simulation was conducted on β-Lactamase and its docked complexes with selected compounds at 300 K. These simulations utilized the GROMOS 54A7 force field (Schuler et al., 2001) within the GROMACS 2020 beta simulation suite (Van Der Spoel et al., 2005). This force field was chosen due to its compatibility with the GROMACS suite its enhanced performance in capturing protein-ligand interactions, and its successful application in similar enzyme-inhibitor studies (Mohammad et al., 2020a; Mohammad et al., 2020b). The topology parameters for the selected compounds were obtained from the Automated Topology Builder (ATB) server (Malde et al., 2011) and incorporated into the protein topology to create the protein-ligand complexes. The complexes were then placed in a cubic box of water where counterions were added to neutralize the system (Mark and Nilsson, 2001). Energy minimization was carried out using the steepest descent approach. The simulation was performed under a constant volume with periodic boundary conditions and a constant pressure of 1 bar. The final MD run involved a 500 ns simulation, and the resulting trajectories were analyzed with the GROMACS toolset. This analysis focused on assessing the stability of the β-Lactamase-ligand complexes and included various parameter calculations to monitor structural changes and variations within the complexes. In addition to the primary 500 ns MD simulations, we performed another independent 500 ns replica simulations for each system (Aristospan-β-lactamase and SM23-β-lactamase) to evaluate the reproducibility of the structural dynamics and interactions. The same parameters and force field were used as in the original simulations. The results were analyzed for structural dynamics, compactness, and hydrogen bond interactions.

### 2.7 Essential dynamics

To analyze the conformational flexibility, atomic motions, and structural stability of β-Lactamase and its docked complexes, we performed the principal component analysis (PCA) by using the simulated trajectories (Sittel et al., 2014). The free energy landscape (FELs) for β-Lactamase and its docked complexes were also generated, which allows the survey of the conformations of proteins in the immediate vicinity of their native states (Papaleo et al., 2009). The FEL helps study the flexibility and the conformation of β-Lactamase before and after compound binding.

## 3 Result and discussion

### 3.1 Molecular docking screening

The start of our study involved the use of molecular docking to investigate a database of 3,648 small molecule drugs approved by the FDA for β-Lactamase. Using a powerful virtual screening protocol allowed us to screen across the entire dataset in a short time, identifying these compounds with promising binding affinities for β-lactamase. Next, we assessed ligand efficiency among top-ranked compounds to identify better binders for β-Lactamase. From the virtual screening analysis, 10 standout candidates were chosen from 3,648 compounds, which gave notable binding scores towards β-lactamase estimated between −9.7 and −11.3 kcal/mol (Table 1). Remarkably, all the identified hits showed a higher docking score for β-Lactamase than the control inhibitor, SM23, with an affinity of −6.7 kcal/mol. This finding indicates that these compounds could be more effective binding partners for β-Lactamase, highlighting their potential as promising candidates against β-Lactamase.

**TABLE 1:** List of screened hits and their binding affinities toward β-Lactamase.

S. No.	*Drug*	Binding Free Energy (kcal/mol)	pKi	Ligand Efficiency (kcal/mol/non-H atom)	Torsional Energy
1	Rifaximin	−11.3	8.29	0.1982	2.1791
2	Temoporfin	−10.7	7.85	0.2058	2.4904
3	Tirilazad	−10.4	7.63	0.2261	1.8678
4	Ergotamine	−10.3	7.55	0.2395	1.5565
5	Ponatinib	−9.9	7.26	0.2538	2.1791
6	Bisdequalinium	−9.8	7.19	0.2227	0
7	Perflunafene	−9.8	7.19	0.35	0
8	Linopirdine	−9.7	7.11	0.3233	1.5565
9	Flunarizine	−9.7	7.11	0.3233	1.8678
10	Aristospan	−9.7	7.11	0.2553	2.1791
11	SM23	−6.9	5.06	0.2654	2.4904

### 3.2 Drug profiling and PASS analysis

After the docking analysis, drug profiling was performed to investigate the drug uses and their biological activities. Here, Aristospan stands out as a promising molecule with appropriate drug profiles and binding potential. Further, a PASS analysis was performed to identify all possible biological characteristics of Aristospan that are associated with β-Lactamase complexities. The PASS analysis of the biological activities of Aristospan produced properties similar to those of the reference inhibitor, SM23. Aristospan has favorable PASS properties, indicating a promise of repurposed drug application in bacterial infections. Aristospan showed predictions for antiinflammatory, antiallergic, antiasthmatic, antipruritic, allergic, and antipsoriatic activities with Pa values from 0.985 to 0.795 (Table 2). Elevated Pa values denote that there is a higher probability that the Aristospan has activity in an antibacterial context. The control inhibitor, SM23, exhibited as a β-Lactamase inhibitor, thus validating the PASS prediction. We also performed an ADMET (absorption, distribution, metabolism, excretion, and toxicity) analysis for the screened compounds. The results demonstrate favorable drug-likeness and bioavailability profiles, further supporting the therapeutic potential of Aristospan without any toxic patterns. The study provided support for the potential development of Aristospan into repurposed therapeutic products against β-Lactamase-associated drug resistance.

**TABLE 2:** Screened compounds and their predicted biological activities along with their ADME profiles. Both compounds are non-toxic in hepatotoxicity and AMES toxicity predictions.

S. No.	Molecule	Pa	Pi	Biological Activity	ADME profiles			
					Absorption	Distribution	Metabolism	Excretion
1	Aristospan	0.985	0.003	Antiinflammatory	Bioavailable	BBB non-penetrable	CYP non-inhibitor	OCT2 substrate
		0.972	0.003	Antiallergic				
		0.963	0.004	Antiasthmatic				
		0.938	0.000	Antipruritic, allergic				
		0.795	0.004	Antipsoriatic				
6	SM23	0.657	0.002	β-Lactamase inhibitor	Bioavailable	BBB non-penetrable	CYP non-inhibitor	OCT2 non-substrate
		0.461	0.029	Antiinfective				
		0.520	0.126	Antieczematic				
		0.381	0.035	Antibacterial				
		0.389	0.046	Antipsoriatic				

### 3.3 Interaction analysis

**FIGURE 1:**
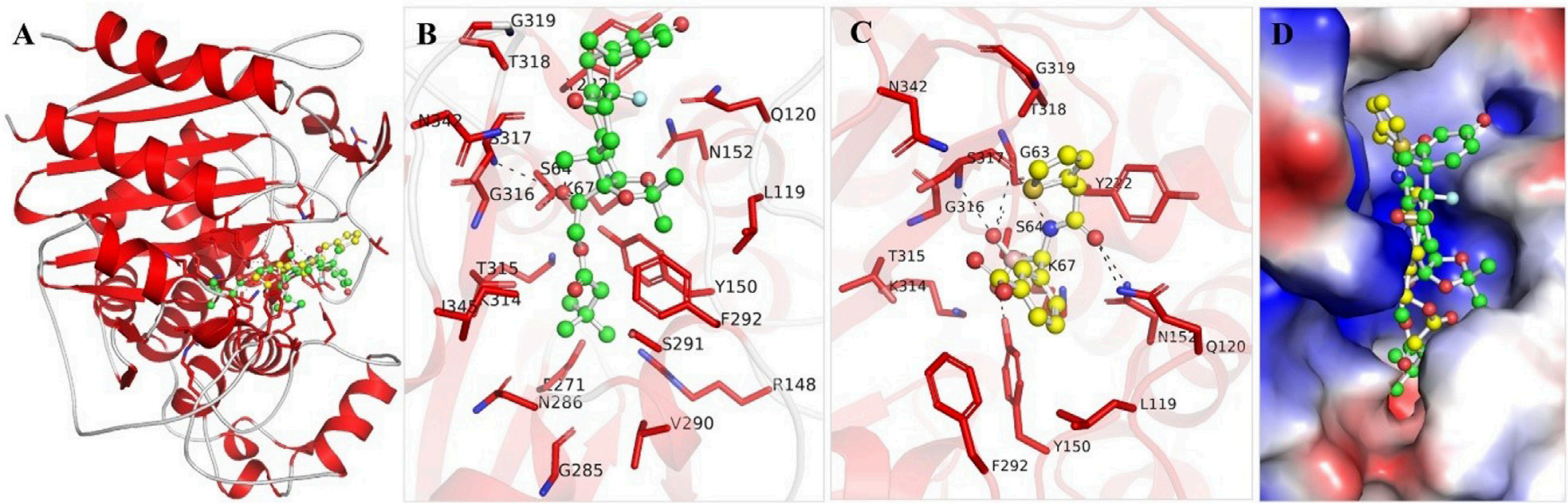
Structural representation of β-Lactamase with docked molecules. **(A)** Cartoon representation of β-Lactamase with Aristospan (green) along with the reference inhibitor SM23 (yellow). **(B)** β-Lactamase binding site residues interacting with Aristospan. **(C)** β-Lactamase binding site residues interacting with SM23. **(D)** Surface potential view of β-Lactamase binding pocket occupied by Aristospan and SM23.

The binding mechanisms of the selected molecules from the docking study were thoroughly analyzed. Using PyMOL and Discovery Studio Visualizer, we conducted an in-depth interaction analysis of Aristospan and SM3. All possible docked conformations for Aristospan and the β-Lactamase reference inhibitor, SM23, were extracted from the docking output files. This analysis focused on binding poses that interacted specifically with the binding site residues of β-Lactamase. Aristospan and SM23 emerged as prominent candidates due to their ability to form hydrogen bonds with important amino acids within the β-Lactamase binding pocket (Figure 1). We found that Aristospan and SM23 preferentially bind to β-Lactamase (Figure 1A). Aristospan showed various close interactions with the binding site of β-Lactamase (Figure 1B). It showed several similar interactions as SM23 with the β-Lactamase binding pocket (Figure 1C). Both compounds are directly bound deep inside the binding pocket of β-lactamase (Figure 1D). Overall, interaction analysis indicated that Aristospan has high potential as a β-lactamase binder and can be exploited as a repurposed therapeutic molecule after required validation.

**FIGURE 2:**
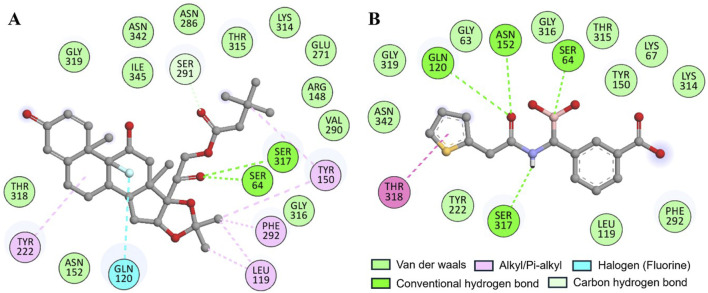
Two-dimensional residual representation of β-Lactamase interacting with **(A)** Aristospan and **(B)** SM23.

The docking of both compounds within the β-Lactamase binding pocket was extensively evaluated for their interactions with functionally important amino acids (Figure 2). The analysis demonstrated that both Aristospan and the control inhibitor SM23 interact with the β-Lactamase binding site region. Aristospan binds to important amino acids within the binding pocket, forming hydrogen bonds with Ser64 and Ser317, among other important residues (Figure 2A). Similarly, SM23 establishes four hydrogen bonds with Ser64, Gln120, Asn152, and Ser317, along with several other important amino acids in the binding site (Figure 2B). The common interactions of both compounds with β-Lactamase suggest that Aristospan may have potential as an inhibitor of β-Lactamase activity.

### 3.4 MD simulations

MD simulation is a widely accepted computation method for drug discovery where how biological structure responds at an atomic level is observed (Shamsi et al., 2019). It gives better details regarding the kinetic properties of proteins, which is very important, aiming at the functional model and the interaction with possible drug molecules (Shamsi et al., 2024). In addition to the initial 500 ns all-atom full MD simulations, we performed independent replica simulations for another 500 ns each to further validate the conformational dynamics and stability of the β-Lactamase-Aristospan and β-Lactamase-SM23 complexes. The results of both the primary and replica simulations were compared by evaluating several structural indices over time as described in the subsequent sections (Table 3).

**TABLE 3:** Different systematic MD parameters and their estimated average values.

System	RMSD (nm)	RMSF (nm)	*R*g (nm)	SASA (nm^2^)	#H-bonds
β-Lactamase	0.16	0.08	1.98	154.57	249
β-Lactamase-Aristospan	0.16	0.09	1.98	155.82	251
β-Lactamase-SM23	0.18	0.10	1.98	157.76	244

#### 3.4.1 Structural deviations and compactness

**FIGURE 3:**
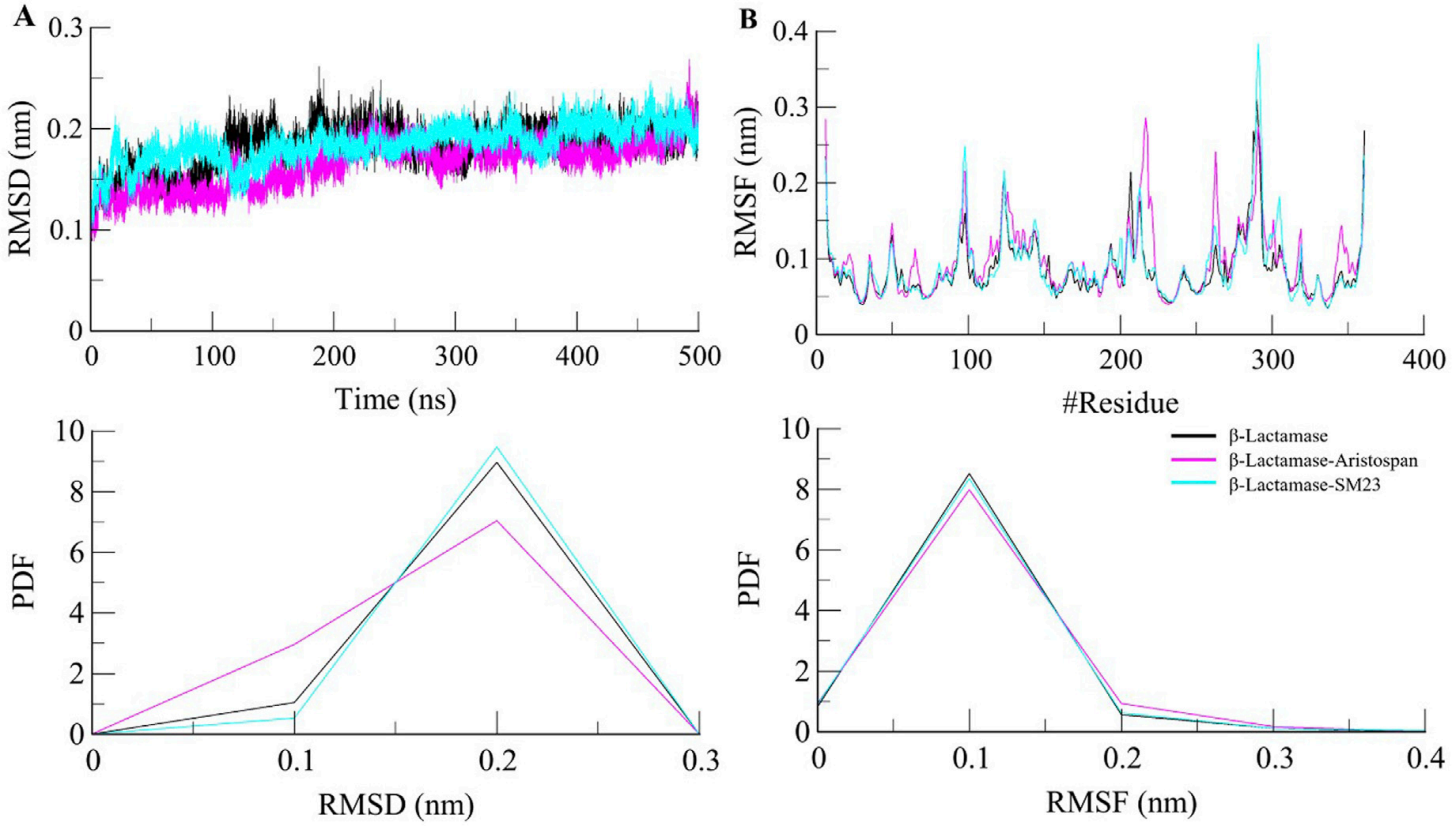
Structural dynamics of β-Lactamase upon Aristospan and SM23 binding. **(A)** RMSD plot of β-Lactamase in complex with Aristospan and SM23. **(B)** Residual fluctuations of β-Lactamase before and after Aristospan and SM23 binding. The dotted boxes showed the major conformational changes in different regions of β-Lactamase after molecules binding.

Structural deviations and fluctuations in a protein molecule have been evaluated by using the root-mean-square deviation (RMSD) (Maiorov and Crippen, 1994). During the simulations, we calculated the time evolution of RMSD values for β-Lactamase and the complex formations of β-Lactamase-Aristospan, as well as β-Lactamase-SM23, and obtained the average RMSD of 0.16 nm, 0.16 nm, and 0.18 nm, respectively. The estimated RMSD values of the β-Lactamase and β-Lactamase-SM23 complexes are equal, which indicates that the protein has reached stability for the entire trajectory as compared to the β-Lactamase-Aristospan complexes (Figure 3A). The resultant RMSD graph indicates that the conformation of the β-Lactamase-Aristospan complex showed reduced distribution and remained stable during the simulation period. This small reduction in the RMSD showed higher stability of the complex (Figure 3A, lower panel). To ensure the reliability and reproducibility of the observed structural dynamics, we performed independent replica simulations of 500 ns for each system (Supplementary Figures S2, S3). The results showed almost identical structural dynamics of β-Lactamase in both cases, confirming the robustness of the simulations (Supplementary Figure S2A). The result implies that the RMSD values are relatively stable and evenly distributed during the simulation.

To investigate further the residual flexibility in free β-Lactamase and its complexes on the interaction with Aristospan and SM23, we calculated the mean fluctuation of all the residues represented as an RMSF plot (Figure 3B). The RMSF chart reveals various amounts of residual oscillations in different parts of β-Lactamase. Interestingly, in the case of β-Lactamase-ligand, these variations are observed to move similarly as free β-Lactamase from the N- to the C-terminal region. As a result, the conformation of the β-Lactamase-Aristospan appeared to be stable in the RMSF analysis. However, when the protein was bound to Aristospan, a minor increase in residual fluctuations was detected in some areas, which suggests increased fluctuations in particular loops. The were a few increased conformational changes in different loop regions of β-Lactamase, S55-G63, R211-L217, E263, and N340-P344. These changes were minimized after the binding of SM23 with an increased loop flexibility in the region of Q293-Q309. The RMSF plot of the RMSF values depicted a very stable pattern of the RMSF that has been experienced after the ligands have bound to the protein, hence displaying a very stable system of the protein-ligand complexes (Figure 3B, lower panel). The results from the replica simulation also showed almost identical residual fluctuations in β-Lactamase in both cases (Supplementary Figure S2B). In addition to the primary MD simulations, the independent 500 ns replica simulations reinforced the observation that Aristospan forms a stable complex with β-lactamase.

**FIGURE 4:**
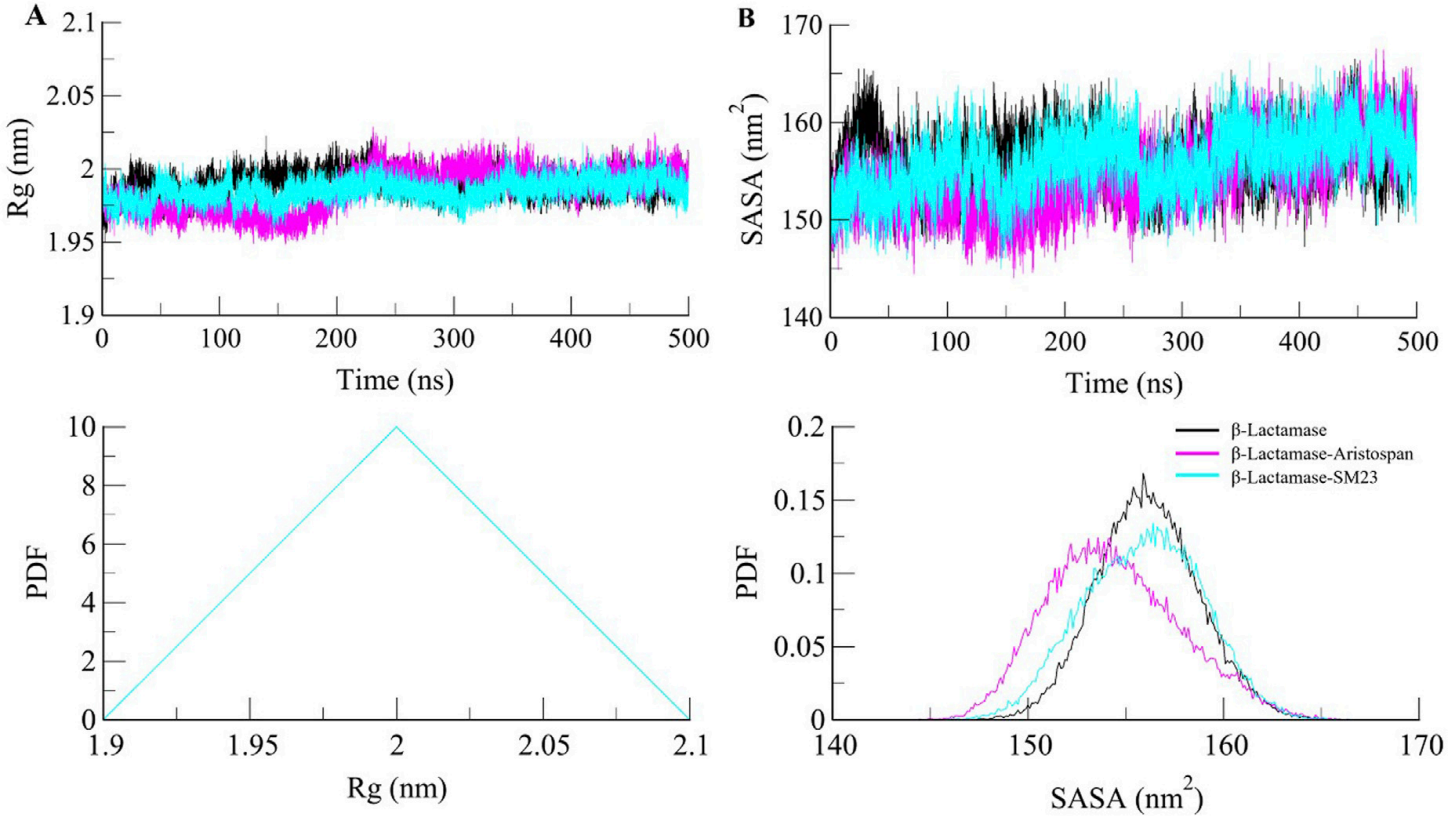
Structural compactness of β-Lactamase upon Aristospan and SM23 binding. **(A)** Time evolution of radius of gyration (*R*g) and **(B)** SASA of β-Lactamase and its docked complexes.

β-Lactamase conformational stability was determined before and after binding to Aristospan and SM23 through the determination of the radius of gyration (*R*g) for both conditions. The average *R*g values calculated for the β-Lactamase in its free state and after the interaction with Aristospan and SM23 were at 1.99 nm, 1.98, and 1.98 nm, respectively. From the *R*g plot analysis, it can be concluded that the fold of β-Lactamase remained compact upon binding to Aristospan and SM23 (Figure 4A). Overall, the study showed that the *R*g values remained relatively constant and balanced throughout the simulation, which confirmed the reliability of the complexes (Figure 4A, lower panel). Based on the average SASA values obtained, β-Lactamase and the β-Lactamase-Aristospan and β-Lactamase-SM23 complexes had mean SASA values of 156.8 nm^2^, 154.4 nm^2^, and 155.5 nm^2^, respectively (Figure 4B). This trend of equilibration was also evident in the SASA plot of all three systems of the protein. A slight decrease in the average SASA values of β-Lactamase-Aristospan indicates that the conformation of β-Lactamase becomes more compact upon binding with Aristospan and SM3 (Figure 4B, lower panel). The results from the replica simulations also showed similar structural compactness of β-Lactamase before and after Aristospan and SM23 binding (Supplementary Figure S2C, D). The structural compactness observed in the replica simulations were consistent with the original simulations, further validating the stability and binding efficiency of Aristospan compared to SM23.

#### 3.4.2 Dynamics of hydrogen bonds

**FIGURE 5:**
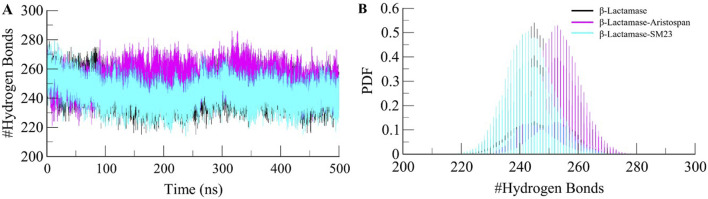
Dynamics of intramolecular hydrogen bonds. **(A)** Time evolution of hydrogen bonds formed intra-β-Lactamase before and after Aristospan and SM23 binding. **(B)** The probability distribution function (PDF) of the hydrogen bonds.

To further obtain a clearer understanding of the stability of β-Lactamase both before and after its binding with Aristospan and SM23, we carefully studied the temporal changes in the hydrogen bonds that developed during the simulation. The detailed analysis of intramolecular hydrogen bond counts in the structure of β-Lactamase showed that it formed an average of 245 hydrogen bonds before the binding of Aristospan and SM23, and it was slightly changed to 253 and 243 after the binding of these compounds, respectively (Figure 5A). To get a better insight into the hydrogen bond dynamics, we also plotted the probability distribution function (PDF) of the data (Figure 5B). The intramolecular hydrogen bonding indicated that the β-Lactamase structure was stable and compact during the entire simulations. The replica simulation also demonstrated similar hydrogen bond dynamics in the β-Lactamase, further supporting the findings from the initial simulations (Supplementary Figure S3D).

**FIGURE 6:**
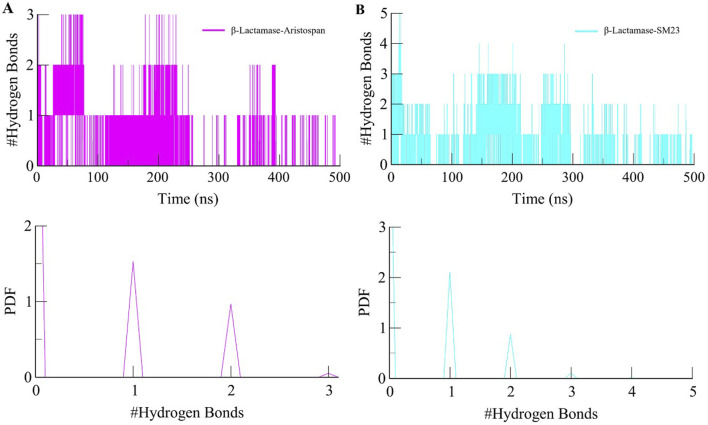
Dynamics of intermolecular hydrogen bonds. **(A)** The time evolution plot shows the formation of intermolecular hydrogen bonds between β-Lactamase and Aristospan. **(B)** The time evolution plot shows the formation of intermolecular hydrogen bonds between β-Lactamase and SM23. Lower panels showed PDF plots of hydrogen bonds.

Studying intermolecular hydrogen bonds in a protein-ligand complex is a powerful approach to understanding the dynamics and stability of these complexes at an atomic level (Bitencourt-Ferreira et al., 2019). We looked at the stability of the intermolecular hydrogen bonds in the bound complexes of β-Lactamase and selected compounds (Figure 6). The study also revealed that the complexes of β-Lactamase-Aristospan and β-Lactamase-SM23 formed 1-3 and 1-5 hydrogen bonds, respectively (Figures 6A, C). The result showed that 1-2 hydrogen bonds were stable and less likely to change during the simulation. The PDF values also revealed a higher degree of order in the complexes' hydrogen bond at one (Figure 6, lower panels). The hydrogen bonds are seen to have a significant contribution towards the stability of both the β-Lactamase-Aristospan and β-Lactamase-SM23 complexes. Furthermore, the analysis of Aristospan and SM23 revealed that these molecules maintain their positions in the protein throughout the simulations, which also confirms the stability of both complexes. The replica simulation also demonstrated similar hydrogen bond dynamics in the β-Lactamase-Aristospan and β-Lactamase-SM23 complexes, further supporting the findings from the initial simulations (Supplementary Figure S3B, C).

### 3.5 Secondary structure analysis

**FIGURE 7:**
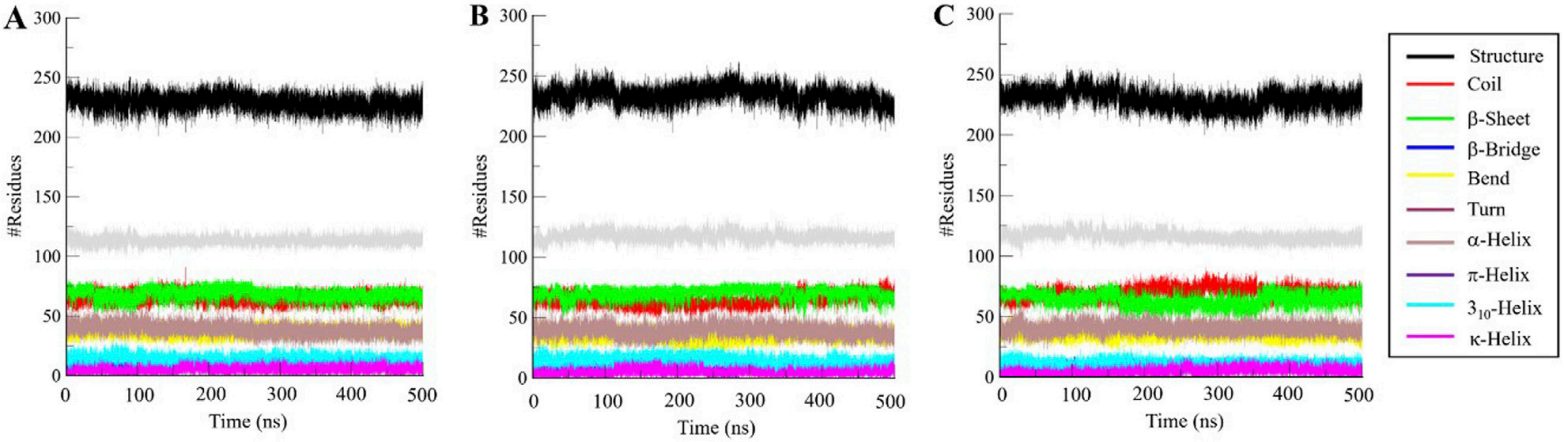
Dynamics of secondary structure elements in **(A)** free β-Lactamase, **(B)** β-Lactamase-Aristospan, and **(C)** β-Lactamase-SM23. The secondary structure content in β-Lactamase is the sum of α-helix, β-sheet, β-bridge, and turns.

We went further deeper in analyzing the time evolution of structural elements to determine the impact of Aristospan and SM23 binding on the secondary structure of β-Lactamase (Figure 7). The result showed that there was significant stability in the secondary structure of free β-Lactamase during the simulation (Figure 7A). Also, the secondary structure makeup of β-Lactamase did not change significantly after binding with Aristospan and SM23 (Figures 7B, C). These secondary structure elements are also preserved and enhance the stability of native protein conformation after the binding of Aristospan and SM23 (Table 4). The analysis has led to the conclusion that the changes to the secondary structure of β-Lactamase after binding to Aristospan and SM23 were comparatively small, which supports the stability of the complexes formed with β-Lactamase-Aristospan and β-Lactamase-SM23.

**TABLE 4:** The number of residues participating in the secondary structure elements of β-Lactamase before and after Aristospan and SM23 binding.

Element	β-Lactamase	β-Lactamase-Aristospan	β-Lactamase-SM23
Coil	65	65	69
β-sheet	69	70	64
β -bridge	8	8	9
Bend	37	34	35
Turn	39	39	41
α-helix	113	117	116
α-helix	6	4	6
3_10_-helix	14	14	11
κ-helix	5	5	5

### 3.6 Principal component analysis

**FIGURE 8:**
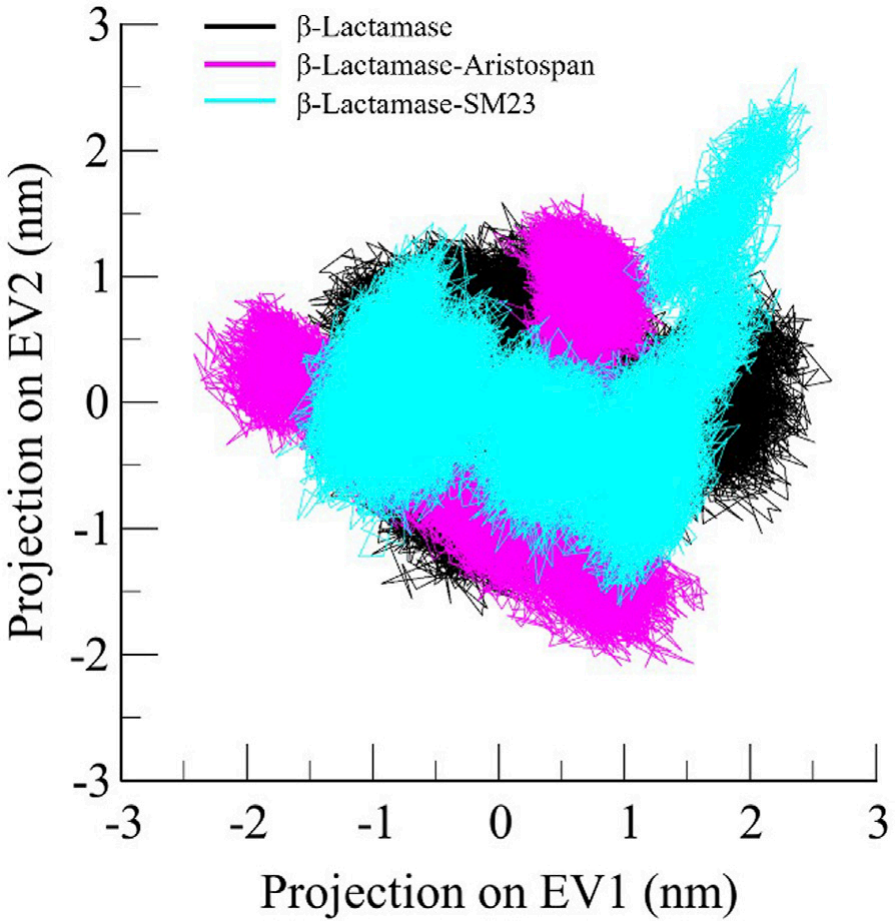
Principal component analysis showed 2D projections of trajectories of β-Lactamase, β-Lactamase-Aristospan, and β-Lactamase-SM23.

Principal component analysis is a widely exploited method for identifying the main trends in protein motions (Sittel et al., 2014). It helps visualize the system dynamics by compressing the configurational space and, thus, helps model protein motion through the essential degrees of freedom. As for the present study, PCA was used to identify conformational variations in the free form of β-Lactamase and its complexes with Aristospan and SM23. The essential dynamics method was applied to study the overall motion of these systems. The conformational flexibility of free β-Lactamase and the β-Lactamase-Aristospan/SM23 complexes was well captured in the essential subspace, as shown in Figure 8, in terms of β-Lactamase’s Eigen Vectors (EV-1 and EV-2) based on the *C*α atoms. Notably, compared to the other systems, the β-Lactamase-Aristospan complex exhibited slightly higher stability while remaining in the same conformational state as the free β-Lactamase. At the same time, the β-Lactamase-SM23 complex showed higher conformational projections at EV2 compared to the free β-Lactamase and β-Lactamase-Aristospan complex. Overall, the PCA showed that the binding of Aristospan and SM23 caused some conformational alterations in β-Lactamase without any major shift during the simulations.

### 3.7 Free energy landscape analysis

**FIGURE 9:**
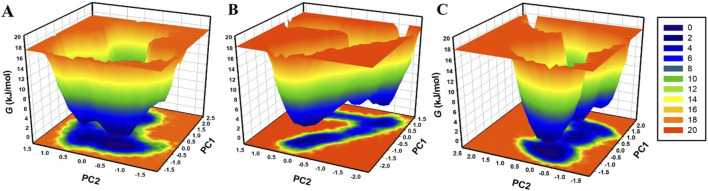
The free energy landscapes of **(A)** free β-Lactamase, **(B)** β-Lactamase-Aristospan, and **(C)** β-Lactamase-SM23.

The FEL analysis provides a detailed picture of the stability of the protein-ligand binding and reveals the possible transition states and metastable conformations (Papaleo et al., 2009). It plays a crucial role in the current drug discovery process, especially in structure-based drug design. For analysis of conformational stability and global minima of β-Lactamase, β-Lactamase-Aristospan, and β-Lactamase-SM23 complexes, we generated FELs using the first two PCs. Figure 9 shows the contoured FELs for the β-Lactamase, β-Lactamase-Aristospan, and β-Lactamase-SM23 complexes from the PCA trajectories. While analyzing these plots, the increased darkness of the color to blue corresponds with the lower energy levels near the native configurations. The analysis showed that β-Lactamase has a single global minimum that is surrounded by two large, localized minima (Figure 9A). When β-Lactamase interacts with Aristospan, the FEL shows the emergence of 2-3 distinct basins, each containing one to two global minima (Figure 9B). This indicates that the β-Lactamase-Aristospan complex exhibits multiple stable conformations with a broader range of energetically favorable states compared to the unbound enzyme. Similarly, the interaction of β-Lactamase with SM23 also results in 2-3 prominent basins, each with one to two global minima (Figure 9C). This observation implies that the β-Lactamase-SM23 complex demonstrates comparable conformational diversity, reflecting various stable binding modes of SM23 within the β-Lactamase binding site. Overall, the FEL findings highlight the dynamic nature of β-Lactamase when interacting with Aristospan and SM23. The presence of multiple basins and global minima in the FELs points to the diverse conformational states that β-Lactamase can adapt to different inhibitors.

### 3.8 Aristospan potential in superbug resistance scenarios

In addition to evaluating the β-Lactamase from *E. coli,* we also examined the binding potential of Aristospan to inhibit β-lactamase enzymes of three different Gram-negative bacteria (*Klebsiella pneumoniae, Neisseria gonorrhoeae,* and *Pseudomonas aeruginosa*) and three Gram-positive bacteria (*Staphylococcus aureus, Bacillus anthracis,* and *Streptococcus pneumoniae*). The results showed that Aristospan showed appreciable binding affinities and bound more favorably to β-lactamases from these organisms compared to the control inhibitor SM23 tested against *E. coli* β-lactamase (Table 5). This broad-spectrum binding potential of Aristospan against both Gram-negative and Gram-positive bacteria indicates its possible use as a versatile β-lactamase enzyme inhibitor, which is very important in antibiotic resistance. The binding of Aristospan to β-lactamases in various types of bacteria makes it a potential candidate in the fight against superbug infections since both Gram-negative and Gram-positive bacteria are known to develop multidrug resistance. Based on the rising trends of antibiotic resistance among these bacterial categories, our findings indicate that Aristospan could be a significant solution to the global problem of superbug resistance. Since Aristospan has the ability to inhibit β-lactamases from organisms that are associated with different resistant infections, it has the potential to be used as a broad-spectrum repurposed therapeutic agent in the treatment of resistant bacterial infections.

**TABLE 5:** The binding affinities and ligand efficiency of Aristospan toward β-Lactamase from different Gram-positive and Gram-negative bacteria.

Bacterium type	Organism	Binding Free Energy (kcal/mol)	pKi	Ligand Efficiency (kcal/mol/non-H atom)
Gram-Negative	*Klebsiella pneumoniae*	−8.8	6.45	0.2316
	*Neisseria gonorrhoeae*	−7.7	5.65	0.2026
	*Pseudomonas aeruginosa*	−7.5	5.5	0.1974
Gram-Positive	*Staphylococcus aureus*	−7.7	5.65	0.2026
	*Bacillus anthracis*	−7.9	5.79	0.2079
	*Streptococcus pneumoniae*	−7.1	5.21	0.1868

## 4 Conclusion

This study sheds light on the drug repurposing against β-Lactamase as a viable strategy of therapeutic development while explicating the importance of finding efficient inhibitors. Here, using a structure-based drug discovery strategy, we proposed Aristospan as a promising repurposed molecule that possesses an appropriate drug profile and appreciable high binding affinity to β-Lactamase enzymes. The broad-spectrum inhibitory potential of Aristospan positions it as a versatile agent in combating superbug resistance. The results obtained in this study are supported by appropriate pharmacological characteristics, binding affinity, and high stability of Aristospan in complex with β-Lactamase. The study reaffirms that Aristospan possesses the necessary attributes to act as a promising beginning structure for the development of other relevant β-Lactamase inhibitors. The study provides important insight into the promising future of Aristospan as a repurposed drug molecule and highlights the potential of a structure-guided drug discovery strategy. Thus, the synergy of molecular information and computational modeling has opened what may prove to be a fruitful direction in the development of drugs for β-Lactamase. In this regard, the present study set up a background for additional research and improvements that may ultimately lead to the development of effective therapies against drug resistance to antibacterial diseases, especially for both Gram-positive and Gram-negative superbugs.

Supplementary Figure S1: Binding pose of SM23 in complex with β-Lactamase. Green and cyan elements show co-crystallized and docked SM23 with β-Lactamase in the grey, respectively. The structural coordinates for the β-Lactamase-SM23 complex were obtained from the RCSB-Protein Data Bank, PDB ID: 5CHJ. Supplementary Figure S2: Replica simulation showed similar structural dynamics and compactness of β-Lactamase upon Aristospan and SM23 binding. (A) RMSD plot of β-Lactamase in complex with Aristospan and SM23. (B) Residual fluctuations of β-Lactamase with Aristospan and SM23. (C) Time evolution of radius of gyration (*R*g) and (D) SASA of β-Lactamase and its docked complexes. Supplementary Figure S3: Replica simulation showed similar dynamics of hydrogen bonds in β-Lactamase and its docked complexes with Aristospan and SM23. (A) Time evolution of hydrogen bonds formed intra-β-Lactamase before and after Aristospan and SM23 binding. (B) The time evolution plot shows the formation of intermolecular hydrogen bonds between β-Lactamase and Aristospan. (C) The time evolution plot shows the formation of intermolecular hydrogen bonds between β-Lactamase and SM23.

## Data Availability

The original contributions presented in the study are included in the article/Supplementary Material, further inquiries can be directed to the corresponding authors.
