# Supplementary material for: Structure-guided drug repurposing identifies aristospan as a potential inhibitor of β-lactamase: insights from virtual screening and molecular dynamics simulations
Source: Front Pharmacol. 2024 Nov 6;15:1459822. doi: 10.3389/fphar.2024.1459822 (PMC11576302; doi:10.3389/fphar.2024.1459822)
Supplement: Supplementary file 1 [file Presentation1.PPTX]

## Slide 1
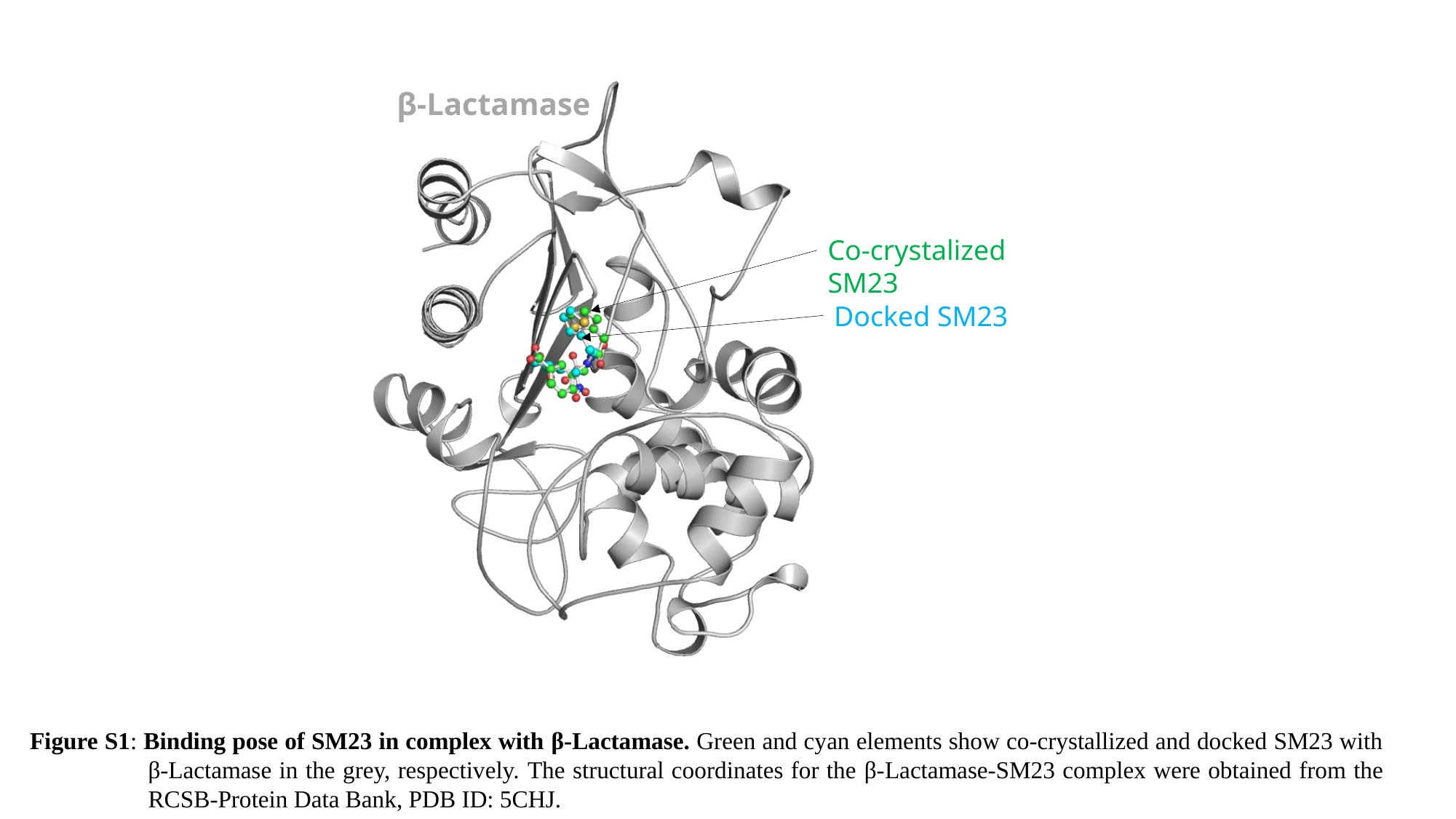

β-Lactamase
Co-crystalized SM23
Docked SM23
Figure S1: Binding pose of SM23 in complex with β-Lactamase. Green and cyan elements show co-crystallized and docked SM23 with β-Lactamase in the grey, respectively. The structural coordinates for the β-Lactamase-SM23 complex were obtained from the RCSB-Protein Data Bank, PDB ID: 5CHJ.

## Slide 2
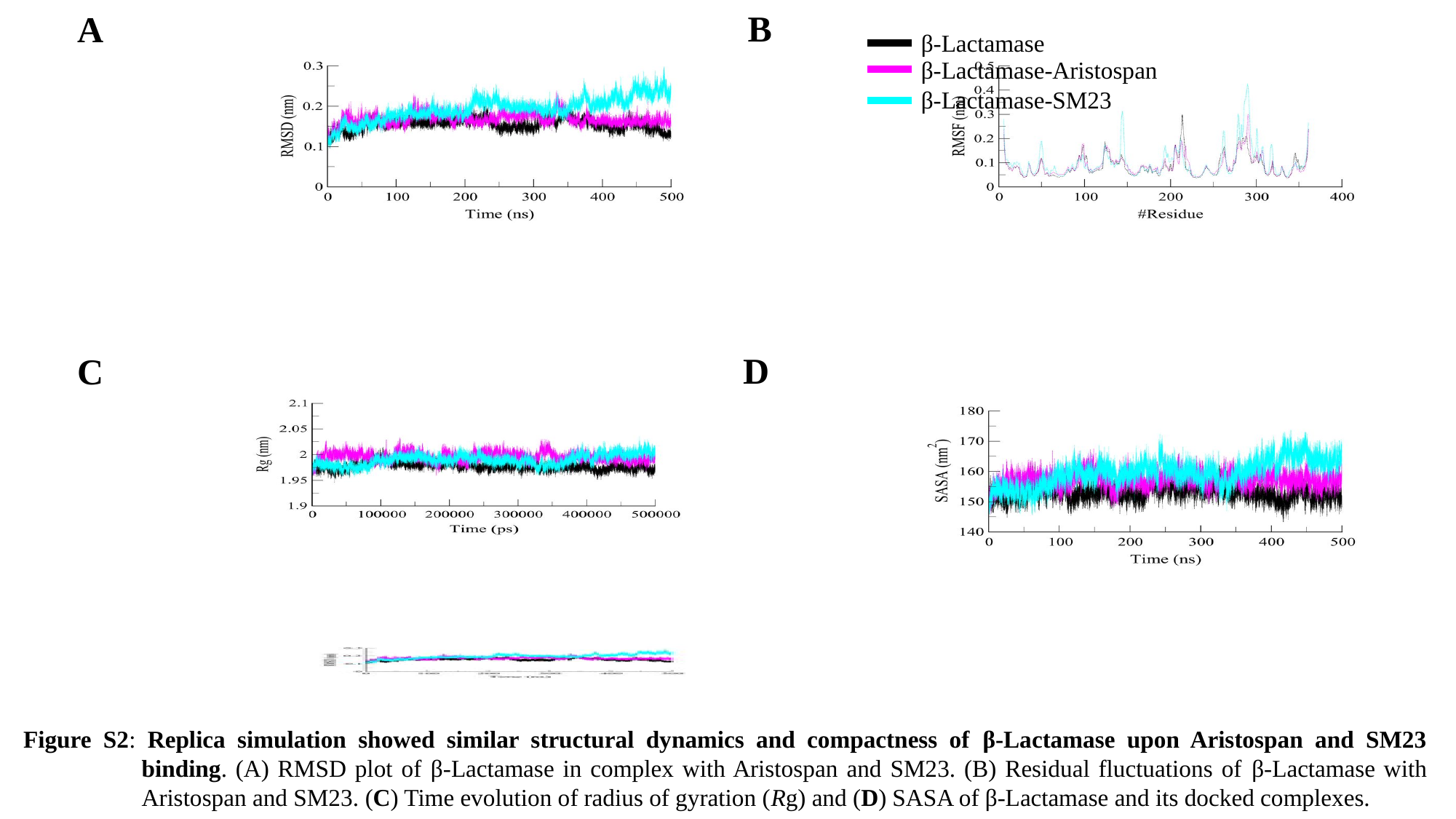

B
A
β-Lactamase
β-Lactamase-Aristospan
β-Lactamase-SM23
D
C
Figure S2: Replica simulation showed similar structural dynamics and compactness of β-Lactamase upon Aristospan and SM23 binding. (A) RMSD plot of β-Lactamase in complex with Aristospan and SM23. (B) Residual fluctuations of β-Lactamase with Aristospan and SM23. (C) Time evolution of radius of gyration (Rg) and (D) SASA of β-Lactamase and its docked complexes.

## Slide 3
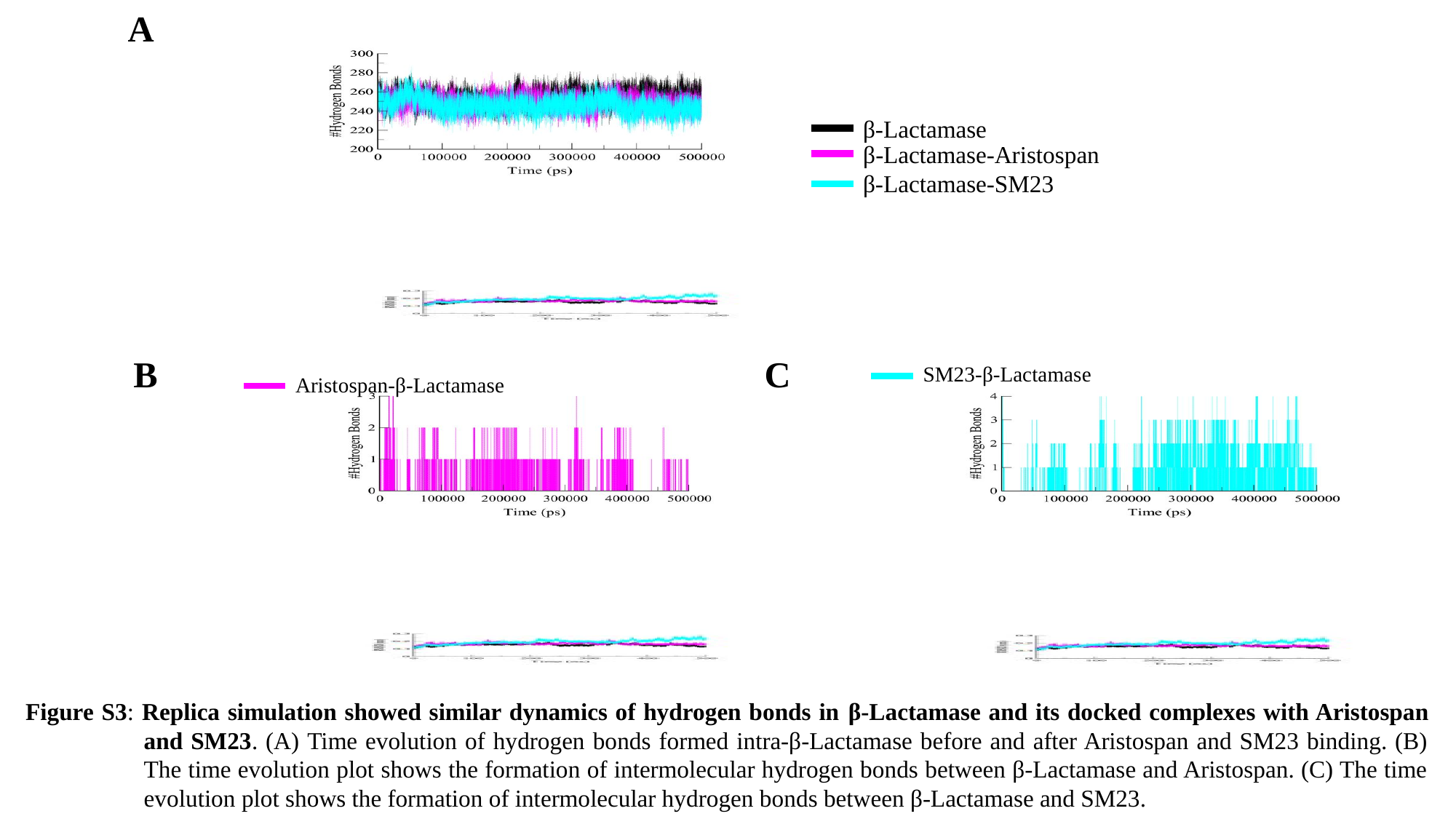

A
β-Lactamase
β-Lactamase-Aristospan
β-Lactamase-SM23
C
B
SM23-β-Lactamase
Aristospan-β-Lactamase
Figure S3: Replica simulation showed similar dynamics of hydrogen bonds in β-Lactamase and its docked complexes with Aristospan and SM23. (A) Time evolution of hydrogen bonds formed intra-β-Lactamase before and after Aristospan and SM23 binding. (B) The time evolution plot shows the formation of intermolecular hydrogen bonds between β-Lactamase and Aristospan. (C) The time evolution plot shows the formation of intermolecular hydrogen bonds between β-Lactamase and SM23.
